# Identification of Antimicrobial Peptides Isolated From the Skin Mucus of African Catfish, *Clarias gariepinus* (Burchell, 1822)

**DOI:** 10.3389/fmicb.2021.794631

**Published:** 2021-12-20

**Authors:** Hedmon Okella, Hilda Ikiriza, Sylvester Ochwo, Clement Olusoji Ajayi, Christian Ndekezi, Joseph Nkamwesiga, Bruhan Kaggwa, Jacqueline Aber, Andrew Glory Mtewa, Tindo Kevin Koffi, Steven Odongo, Didier Vertommen, Charles Drago Kato, Patrick Engeu Ogwang

**Affiliations:** ^1^Pharm-Biotechnology and Traditional Medicine Centre, Mbarara University of Science and Technology, Mbarara, Uganda; ^2^Department of Biotechnical and Diagnostic Sciences, College of Veterinary Medicine Animal Resources and Biosecurity, Makerere University, Kampala, Uganda; ^3^MRC/UVRI & LSTMH Uganda Research Unit, Entebbe, Uganda; ^4^International Livestock Research Institute, Kampala, Uganda; ^5^Department of Pharmacy, Faculty of Medicine, Gulu University, Gulu, Uganda; ^6^Chemistry Section, Malawi Institute of Technology, Malawi University of Science and Technology, Limbe, Malawi; ^7^Department of Food Science and Technology, Chungnam National University, Daejeon, South Korea; ^8^de Duve Institute and MASSPROT Platform, UCLouvain, Brussels, Belgium

**Keywords:** African catfish, antimicrobial peptides, MIC, skin mucus, Uganda

## Abstract

Antimicrobial peptides (AMPs) constitute a broad range of bioactive compounds in diverse organisms, including fish. They are effector molecules for the innate immune response, against pathogens, tissue damage and infections. Still, AMPs from African Catfish, *Clarias gariepinus*, skin mucus are largely unexplored despite their possible therapeutic role in combating antimicrobial resistance. In this study, African Catfish Antimicrobial peptides (ACAPs) were identified from the skin mucus of African Catfish, *C. gariepinus*. Native peptides were extracted from fish mucus scrapings in 10% acetic acid (v/v) and ultra-filtered using 5 kDa molecular weight cut-off membrane. The extract was purified using C_18_ Solid-Phase Extraction. The antibacterial activity was determined using the Agar Well Diffusion method and broth-dilution method utilizing *Staphylococcus aureus* (ATCC 25923) and *Escherichia coli* (ATCC 25922). Thereafter, Sephadex G-25 gel filtration was further utilized in bio-guided isolation of the most active fractions prior to peptide identification using Orbitrap Fusion Lumos Tribrid Mass Spectrometry. The skin mucus extracted from African Catfish from all the three major lakes of Uganda exhibited antimicrobial activity on *E. coli* and *S. aureus*. Lake Albert’s *C. gariepinus* demonstrated the best activity with the lowest MIC of 2.84 and 0.71 μg/ml on *S. aureus* and *E. coli*, respectively. Sephadex G-25 peak I mass spectrometry analysis (Data are available *via* ProteomeXchange with identifier PXD029193) alongside *in silico* analysis revealed seven short peptides (11–16 amino acid residues) of high antimicrobial scores (0.561–0.905 units). In addition, these peptides had a low molecular weight (1005.57–1622.05 Da) and had percentage hydrophobicity above 54%. Up to four of these AMPs demonstrated α-helix structure conformation, rendering them amphipathic. The findings of this study indicate that novel AMPs can be sourced from the skin mucus of *C. gariepinus*. Such AMPs are potential alternatives to the traditional antibiotics and can be of great application to food and pharmaceutical industries; however, further studies are still needed to establish their drug-likeness and safety profiles.

## Introduction

Infections caused by bacteria are progressively threatening human and animal lives, particularly with the increasing antimicrobial drug resistance (Outterson et al., 2015). As such, a pressing need for fresh approaches to combat infections caused by antibiotic-resistant micro-organisms is highly required. Biologics, particularly native antimicrobial peptides (AMPs) are one of the essential effector molecules in many forms of life, including fish, to fight microbial tissue invasion (Sarmasik, 2002; Huan et al., 2020). Such peptides are essential in the fish innate immunity (Rottmann et al., 1992), especially AMPs in the skin mucus of scale-less fish thriving in often pathogen-dense aquatic environments (Lee et al., 2020). Interestingly, the majority of these peptides have demonstrated broad-spectrum activity, even on bacteria that resist traditional antibiotics (Fuochi et al., 2017; Välimaa et al., 2019; Tiralongo et al., 2020). However, instability alongside hemolytic side effects limits the applicability of most AMPs. One approach to addressing such drawbacks is exploring and developing novel natively existing safe and efficacious AMPs from the pathogen-dense aquatic inhabitants. These can potentially avail and as well improve pharmaceutical leads of value over traditional antibiotics in the era of antimicrobial drug resistance.

In light of the above, fish remains a potential source of AMPs, given that, they are by far the most abundant vertebrates on earth, comprising of 33,932 species recorded worldwide (Froese and Pauly, 2020). Regrettably, only a few fish AMPs (136) have been identified (Wang et al., 2016) as of 3rd July 2021.^1^ The number is even lower in the context of fish mucosal AMPs. The few notable examples of the mucosal AMPs are Pelteobagrin, a 20-residue peptide from yellow catfish, *Pelteobagrus fulvidraco* (Su, 2011); Myxinidin, a 12-residue peptide from hagfish, *Myxine glutinosa* (Subramanian et al., 2009); Histone H2B-derived peptide from Atlantic cod, *Gadus morhua* (Bergsson et al., 2005); SAMP-H1 (a proline-rich histone H1-derived peptide from Atlantic salmon, *Salmo salar*; Luders et al., 2005); Oncorhyncin II, a histone H1-derived peptide from rainbow trout, *Oncorhynchus mykiss* (Fernandes et al., 2004); Hipposin, a 51-residue peptide from Atlantic alibut, *Hippoglossus hippoglossus* (Birkemo et al., 2003); Pleurocidin, a 25-residue peptide from winter flounder, *Pleuronectes americanus* (Cole et al., 1997); Pardaxin, a 33-residue peptide from Moses sole fish, *Pardachirus marmoratus* (Oren and Shai, 1996) among others. The majority of the currently identified AMPs are from marine fish. Emphasis on freshwater fish skin mucus in the African region is far insufficient, with no record of identified peptide class by primary structure in the skin mucus of the African Catfish, *Clarias gariepinus* (Burchell, 1822). Therefore, in an effort to develop novel alternative antibiotic candidates, this study presents the first report on identifying AMPs from the skin mucus of the African catfish, *C. gariepinus*.

## Materials and Methods

### Fish and Mucus Collection

**Figure 1:**
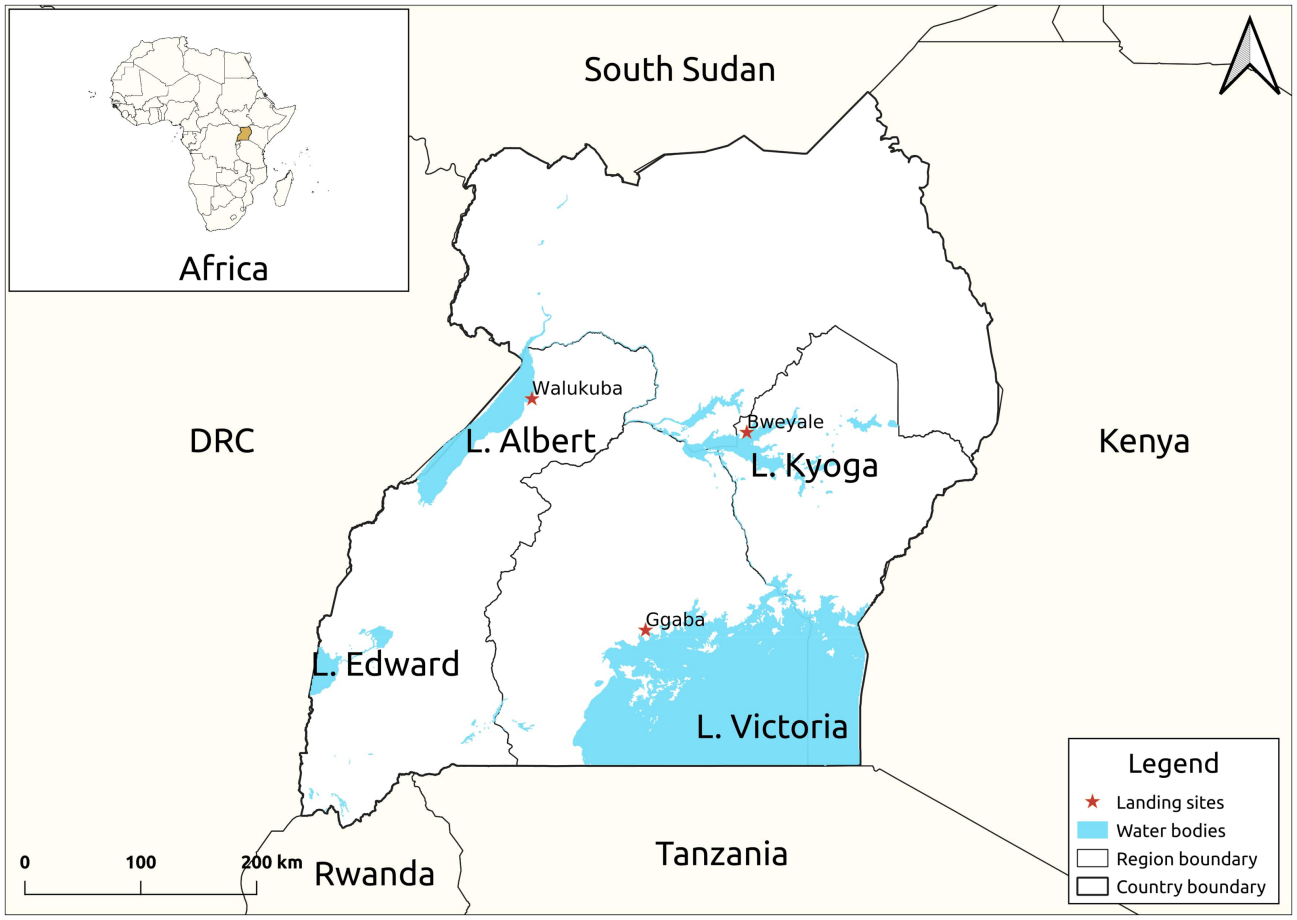
Map of Uganda showing the study sites. The samples were collected from the landing sites of Lake Victoria, Kyoga and Albert.

Twenty-four live mature *C. gariepinus* (Burchell, 1822) in the family *Clariidae* (mean weight 300.50 ± 5.98 g, mean length 30.60 ± 2.11 cm), all in the second growth phase, were sourced from each of the purposively selected three major freshwater lakes [Kyoga (GPS: 1.5876, 33.0494), Victoria (GPS: 0.2578, 32.6375) and Albert (GPS: 1.8481, 31.3819)] of Uganda (Figure 1). Fish in the second growth phase (100–1,500 g) produces substantial quantity of skin mucus even at high stocking density (van de Nieuwegiessen et al., 2009). The fish were then separately transported, later acclimatized and fed *ad labitum* for 7 days on commercial 8 mm Catfish pellets (Kaffiika, Kampala, Uganda). Acclimatization was done in three separate 1,000-litre plastic tanks at the College of Veterinary Medicine, Animal Resources and Biosecurity, Makerere University, Kampala. To optimize the harvest, the fish were starved for 24 h prior to mucus collection. Skin mucus (45 ml) of all the eight fish representing each lake was dorso-laterally scrapped using a sterile plastic spatula and pooled into 50 ml centrifuge tubes (Corning, New York, United States). To avoid contamination from anal and intestinal excreta, scrapping was avoided from the ventral part of the fish. The harvested skin mucus was then lyophilized using a Labconco lyophilizer (Labconco, Kansas, United States) at −104°C and 0.013 millibars to remove excess water and later stored at 4°C for 1 week given its stability between 4 and 25°C.

### Peptide Extraction

Extraction of peptides was carried out using a method adopted from Subramanian et al. (2009), with minor modifications. Briefly, lyophilized skin mucus (0.4 g) was reconstituted in 15 ml of 10% (v/v) acetic acid and heated for 5 min in a boiling water bath at 95°C. The acid mucus mixtures were then homogenized for 1 min using a polytron homogenizer (Kinematica, Malters, Switzerland), Insoluble mucus components were removed by centrifuging at 10,000 × *g* at 4°C for 1 h (Hermle, Wehingen, Germany). The supernatant (14 ml) was then collected into Vivaspin tubes containing a 5 kDa molecular weight cut-off membrane (Sartorius, Gloucestershire, United Kingdom) and centrifuged at 6000 x *g* at 4°C for 10 h to exclude the antimicrobial proteins of high molecular weight present in the mucus. The ultra-filtered peptides with molecular weight below 5 kDa were collected for Solid-Phase Extraction (SPE).

### Isolation of Antimicrobial Peptides by Solid-Phase Extraction

As described by Rana et al. (2018), low molecular weight contaminants were removed from ultra-filtrate by Solid-Phase Extraction. The mucus extracts were applied on disposable SPE C_18_ stationary phase cartridges (50 μm particle size, 60 Å pore diameter, Column Volume 15 ml) containing 5 g sorbent (ThermoScientific, Bellefonte, United States). Columns were conditioned with 30% (v/v) acetonitrile, and subsequently ultra-filtered sample was loaded at a flow rate of 1 ml/min. Only the peptides bind transiently to hydrophobic matrix, leaving contaminants that may confound the peptide extracts to flow through. Bound peptides were mildly washed with deionized water (pH 7.01). Finally, cleaned and concentrated peptides were eluted in 5 ml volume with 70% (v/v) acetonitrile and lyophilized at −104°C and 0.013 millibars. Lyophilization below eutectic temperature of −45°C and 1.67 milibars pressure completely removes acetonitrile that may confound bioactivity of peptide extracts (Harcum, 2008). Later, the antimicrobial activity of the peptides was assessed.

### Bacterial Cell Culturing and Bacterial Susceptibility Testing

*Escherichia coli*, ATCC 25922 and *Staphylococcus aureus*, ATCC25923 were used in this study for antimicrobial activity. These American Type Culture Collection (ATCC) strains were purchased from Thermo Fisher Scientific, Newport, United Kingdom. The choice was guided by the World Health Organization (WHO) Global Priority pathogen listing (Asokan et al., 2019). A Gram-negative human pathogen in the critical priority (*E. coli*) and a Gram-positive pathogen of high priority (*S. aureus*) were selected. Both strains were maintained on Nutrient Agar (Himedia, Mumbai, India). Ciprofloxacin (Medicon, Vadodara, India) was used as a control drug.

### Antimicrobial Activity

The agar well diffusion method described by de Oliveira et al. (1998) was followed. In this method, *S. aureus* ATCC25923 and *E. coli* ATCC25922 were plated on the solid Nutrient agar medium with a concentration of 10^6^ cfu/ml. Pluralities of small wells with a diameter of 6 mm were punched out with a cork borer, and 50 μl of 50 μg/ml laboratory-prepared antibacterial peptides were dispensed into the wells in triplicates. Ciprofloxacin (50 μg/ml) was dispensed in separate wells as the control drug, while 0.01 M Phosphate Buffer Saline (50 μl) was used as negative control, given its inactive role as a non-antimicrobial agent. Besides, complete evaporation of acetonitrile in the SPE dry peptide powder required reconstitution in a solvent (0.01 M PBS) that preserves peptide integrity (Chen et al., 2017). After 24 h of incubation at 37°C, the antibacterial activity of the extracted peptide was determined according to the diameter of the zone of inhibition (mm).

### Determination of Minimum Inhibitory Concentration

Using the broth dilution method, as previously described by Teh et al. (2017), in a 96-well U-shaped cell culture plate (Greiner, Kremsmünster, Austria), 50 μl of Brain Heart Infusion Broth was introduced into six vertical columns. To the first three wells of vertical columns, 0.5 mg/ml extracts of *C. gariepinus* from Lake Victoria, Lake Albert, and Lake Kyoga were each added at 50 μl/well, respectively. The three subsequent columns contained ciprofloxacin (50 μg/ml) as a positive control, 0.01 M PBS as a negative control, and sterile broth. Subsequently, a two-fold serial dilution was performed after which 50 μl of diluted bacterial suspension (1.5 × 10 cell/ml) was added into all wells (except the broth sterility control column) and mixed thoroughly. Micro-dilution was performed in triplicates for each bacterial species. After overnight incubation at 37°C, 50 μl of 6.75 mg/ml Resazurin (Glentham, Wiltshire, United Kingdom) was added to all wells and incubated at 37°C for another 4 h. Color changes were observed and recorded, with microbial growth implied by an irreversible change in the color from the blue of resazurin to pink resofurin. The lowest concentration prior to the color change was considered as the Minimum Inhibitory Concentration (MIC). Skin mucus extract with the lowest MIC was later selected for further purification.

### Sephadex G-25 Gel Filtration Purification of Antimicrobial Peptides

Lake Albert’s *C. gariepinus* extract (SPE lowest MIC across both bacterial strains) was further purified by a Sephadex G-25 gel filtration column (1.2 × 55 cm; Cytiva, Uppsala, Sweden; Neumann et al., 1996). Briefly, 5 ml of the peptides in 0.01 M PBS with a 10 mg/ml concentration were loaded into the column and eluted by deionized water (pH 7.01) at a flow rate of 0.3 ml/min. Deionized water was utilized as eluent of no antimicrobial activity. The fractions were collected at 9-min intervals with a fraction collector, and absorbance was monitored at 280 nm using a *NanoDrop* Spectrophotometer (Thermo Scientific, New York, United States). A wavelength of 280 nm was used since presence of amino acids with aromatic rings implies maximum absorbance peak at 280 nm (Schmid, 2001). The antibacterial activity of each of the peak fractions on *E. coli* and *S. aureus* was evaluated using the broth-dilution method as previously described. The peak fraction with the highest antibacterial activity was lyophilized and shipped to Proteomics platform, de Duve Institute, UClouvain (Belgium) for peptide sequencing by High-Resolution, Accurate-Mass (HR/AM) mass spectrometry.

### Peptide Sequencing by Mass Spectrometry

The molecular mass and the amino acid sequence of peptides in the most active fraction from gel filtration chromatography (Peak I) were determined using the Orbitrap Fusion Lumos Tribrid Mass Spectrometer (Thermo Fisher, United States) at the de Duve Institute Proteomics platform. Freeze-dried sample was reconstituted at 1 mg/ml in distilled water prior to 10 fold dilution in 3% acetonitrile (ACN)/0.1% trifluoroacetic acid (TFA). Later, 2 μl of the peptides was directly loaded onto reversed-phase pre-column (Acclaim PepMap 100, Thermo Scientific, United States) and eluted in backflush mode. Peptide separation was performed using a reversed-phase analytical column equilibrated in 3.5% ACN 0.1% formic acid (FA) in water (Acclaim PepMap RSLC, 0.075 × 250 mm, Thermo Scientific, United States) with a linear gradient of 4–27.5% solvent B (0.1% FA in 80% ACN) for 100 min, 27.5–40% solvent B for 10 min, 40–95% solvent B for 1 min and holding at 95% for the last 6 min at a constant flow rate of 300 nl/min on an Ultimate 3,000 RSLC nanoHPLC system (Thermo Fisher Scientific, Waltham, United States). The peptides were analyzed in positive mode by an Orbitrap Fusion Lumos Tribrid mass spectrometer (ThermoFisher Scientific, Waltham, United States). The peptides were subjected to Nanospray Ionization (NSI) source followed by tandem mass spectrometry (MS/MS) in Fusion Lumos coupled online to the Ultra Performance Liquid Chromatography (UPLC). Intact peptides were detected in the Orbitrap at a resolution of 120,000 within a mass-charge (*m/z*) range from 350 to 1,500. A charge state decision tree was applied for all precursors’ ions prior to MS2 fragmentation. Peptides with charge states +2 and +3 were selected for MS/MS using Higher-energy C-trap dissociation (HCD) setting at 30; ion fragments were detected in the Orbitrap at a resolution of 30,000. For precursors with charge states from +3 up to +8, MS/MS was obtained by Electron-Transfer/Higher-Energy Collision Dissociation (EThcD) fragmentation with supplemental energy set at 30, ion fragments were detected in the Orbitrap at a resolution of 30,000. A data-dependent procedure of MS/MS scans was applied for the top precursor ions above a threshold ion count of 2.0E4 in the MS survey scan with 60.0 s dynamic exclusion. The total cycle time was set to 4 s. The electrospray voltage applied was 2.1 kV. The MS1 spectra were obtained with an Automatic Gain Control (AGC) target of 4E5 ions, and MS2 spectra were acquired with an AGC target of 5E4 ions, maximum injection time was set to auto for both type of scans. The mass spectrometry proteomics data have been deposited to the ProteomeXchange Consortium *via* the PRIDE (Perez-Riverol et al., 2019) partner repository with the dataset identifier PXD029193 and 10.6019/PXD029193.

### Peptide Identification and Database Search

The resulting MS/MS data were processed using *Sequest HT* search engine within *Proteome Discoverer* 2.4 against a custom database containing 102,688 sequences from catfish species (compiled from Uniprot proteins entries with taxonomy ID: 175774 *Bagarius yarrelli*; 310915 *Pangasianodon hypophtalmus*; 7,998 *Ictalurus punctatus*; 35,657 *Clarias microcephalus*; 219,545 *Ameiurus melas*). No enzyme was specified as cleavage enzyme allowing a maximum peptide length of 30 residues, four modifications per peptide *b*- and *y*- ions for HCD fragmentation, *b*- *c*- *z*- and *y*- ions for EThcD fragmentation. The mass error was set to 10 ppm for precursor ions and 0.1 Da for fragment ions. Oxidation on methionine (Met) was considered as variable modification. Peptide matches were filtered using the q-value and Posterior Error Probability calculated by the Percolator algorithm ensuring an estimated false positive rate (FDR) below 5%. The filtered *Sequest HT* output files for each peptide were grouped according to the protein from which they were derived. Using *FASTA* file input, all the identified peptides from different precursor proteins were subjected to a Discriminant Analysis (DA) machine learning algorithm at the Collection of Anti-Microbial Peptides (CAMP_R3_) server^2^ (Waghu et al., 2016), to reveal their antimicrobial probability. The server gives a score on 0 to1 scale, with a score of >0.5 classified as Antimicrobial Peptide (AMP) and those below 0.5 are Non-Antimicrobial Peptide (NAMP). Subsequently, the *FASTA* file sequences of AMPs with the highest score (Antimicrobial score >0.9), were then inputted to a more rigorous and accurate the Deep Learning algorithm cross-validation using *Deep-AmPEP30* server at https://cbbio.online/AxPEP/ (Yan et al., 2020). The server’s default antimicrobial peptide classification cut-off of 0.5 was maintained. Its positive sequence (Product Probability >0.5) is scored as 1, negative sequence (Product Probability <0.5) is 0 and invalid sequence is −1. Peptides with the highest antimicrobial activity were obtained for *in silico* characterization.

### Sequence Characterization

Using the raw sequence files, molecular weights, net charge at pH 7 and percentage hydrophobicity were predicted using the European Bioinformatics Institute (EBI) tool; *EMBOSS PepStats* at https://www.ebi.ac.uk/Tools/seqstats/emboss_pepstats/. Thereafter, the same ACAPs sequences were utilized in the classification of their secondary structures using Profile network prediction HeiDelberg (PHD) software at https://npsa-prabi.ibcp.fr/cgi-bin/npsa_automat.pl?page=/NPSA/npsa_phd.html (Combet et al., 2000). Later, *PEP-FOLD* v3.5, a web-based *de novo* peptide structure prediction tool at https://bioserv.rpbs.univ-paris-diderot.fr/services/PEP-FOLD/ (Thévenet et al., 2012), was utilized to predict the three-dimensional (3D) structure of the peptide with the highest antimicrobial score. Here, *FASTA* files were inputted, 100 simulations run and later, best output models ranked based on sOPEP energies and Apollo predicted melting temperature (t_m_). The prediction was cross-validated using Associative Memory Water mediated and Energy Model (AWSEM) suite at https://awsem.rice.edu/ (Jin et al., 2020) using *FASTA* file input. Quality check was performed on the best modeled structures (AWSEM and *PEP-FOLD* v3.5 modeled) using a web-based Protein Structure Analysis (*ProSA*) tool^3^ (Wiederstein and Sippl, 2007). The ProSA tool foretells the query protein *z*-score, residual energy and thereafter plots local model quality. The ProSA *z*-score equates the query protein *z*-score against those of experimentally validated proteins at the Protein Data Bank (PDB) Library^4^ (Berman et al., 2002). A higher *z*-score value indicates greater similarity.

### Data Analysis

*Tableau* Software *v2019.4* was used to present data. *GraphPad 5.0* statistical package was used to analyze the antimicrobial activity of skin mucus extracts. The Map was drawn in *Quantum GIS V. 3.10* using the GPS coordinates taken from the landing sites during sample collection. The points were mapped on the Uganda shape files retrieved from *Humanitarian Data Exchange v1.43.6* (HDX) web-server.^5^ Statistical software, SPSS *v16.0* was used to compare the means, in which a one-sample *t*-test was performed to determine the significance of the antimicrobial activity of skin mucus extracts of *C. gariepinus* on *E. coli* and *S. aureus*.

### Ethics Statement

Ethical approval to conduct this study was obtained from the Mbarara University of Science and Technology Research Ethics Committee (Approval No. 22/11-18). The study was as well registered and approved by the Uganda National Council for Science and Technology (Approval Reference No. HS449ES).

## Results

### Solid-Phase Extraction

By removing the low molecular weight contaminants, solid-phase extraction remains an essential tool for purifying and concentrating samples. Upon subjecting the most active ultra-filtrate to SPE, there was a general increase in average concentration peptide (<5 kDa) for all the extracts from 15.04 ± 1.20 to 84.09 ± 0.98 mg/ml.

### Antimicrobial Activity

**Figure 2:**
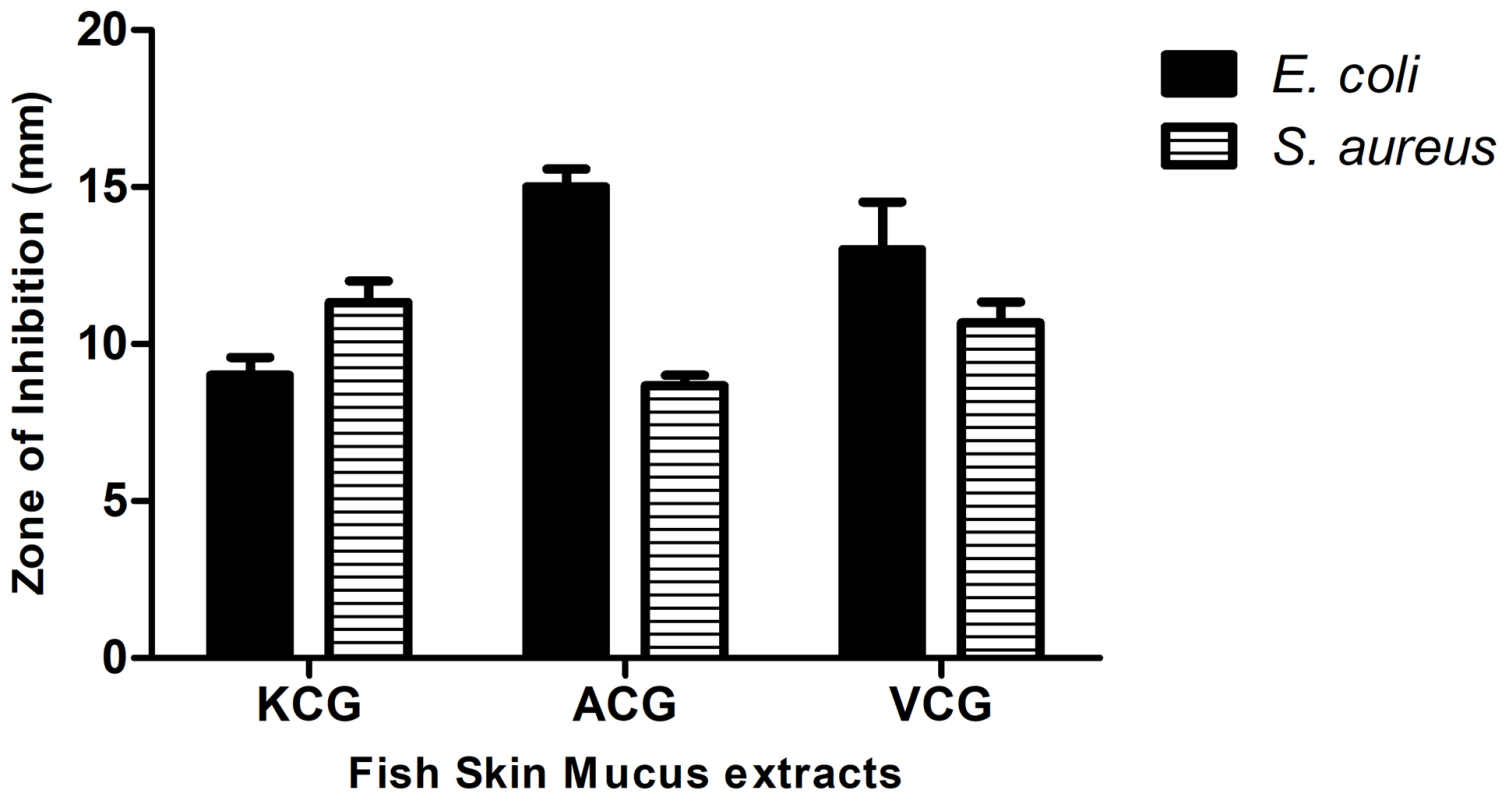
Graph showing Zone of Inhibition of *Clarias gariepinus* mucus extracts from different lakes. VCG-Lake Victoria’s *C. gariepinus* ACG-Lake Albert’s *C. gariepinus* KCG-Lake Kyoga’s *C. gariepinus*. Lake Albert’s *C. gariepinus* skin mucus extracts have the highest Zone of Inhibition (15.00 ± 0.58 mm) on *E. coli*. The highest Zone of Inhibition on *Staphylococcus aureus* was registered on Lake Kyoga’s fish skin mucus extracts (Zone of Inhibition: 11.67 ± 0.67 mm). The image was rendered in *GraphPad 5.0* statistical package.

The skin mucus peptide extract of *C. gariepinus* was found to inhibit the growth of both *E. coli* and *S. aureus* (Figure 2). This zone of inhibition ranged from 9.00 ± 0.58 to 15.00 ± 0.58 mm on *E. coli* and 8.67 ± 0.33 to 11.67 ± 0.67 mm on *S. aureus*. Here, the skin mucus extracts from Lake Albert’s Catfish demonstrated the highest Zone of Inhibition (Zone of Inhibition: 15.00 ± 0.58 mm) on *E. coli*. Both PBS and Acetonitrile did not show any antimicrobial activity. On the other hand, the highest zone of inhibition on *S. aureus* was shown by the Lake Kyoga’s fish skin mucus extracts (Zone of Inhibition: 11.67 ± 0.67 mm). The lowest Zone of Inhibition (8.67 ± 0.33 mm) was registered on *S. aureus*. Similarly, Lake Albert’s SPE extracted peptides demonstrated the highest antimicrobial activity by having the lowest MIC across *E. coli* and *S. aureus* (MIC: 0.71 ± 0.33 and 2.84 ± 0.11 μg/ml on *E. coli* and *S. aureus*, respectively), and was therefore selected for the downstream processes (Table 1). Though not significant (*p* > 0.05), the MIC was much lower on *E. coli* compared to *S. aureus*.

**Table 1:** Minimum Inhibitory Concentration (MIC) of fish skin mucus extract on *E. coli* and *S. aureus*.

Run	MIC (μg/ml)	
	*E. coli*	*S. aureus*
L. Kyoga’s *C. gariepinus*	1.46 ± 0.08	2.92 ± 0.04
L. Victoria’s *C. gariepinus*	0.55 ± 0.02	7.04 ± 0.13
L. Albert’s *C. gariepinus*	0.71 ± 0.33	2.84 ± 0.11
Peak I	0.31 ± 0.16	1.99 ± 0.13
Peak II	ND	ND
Ciprofloxacin	0.50 ± 0.02	0.61 ± 0.04
1 × PBS	ND	ND

*Data are expressed as mean ± SEM and ND, not detected*.

### Sephadex G-25 Gel Filtration Purification of Antimicrobial Peptides

**Figure 3:**
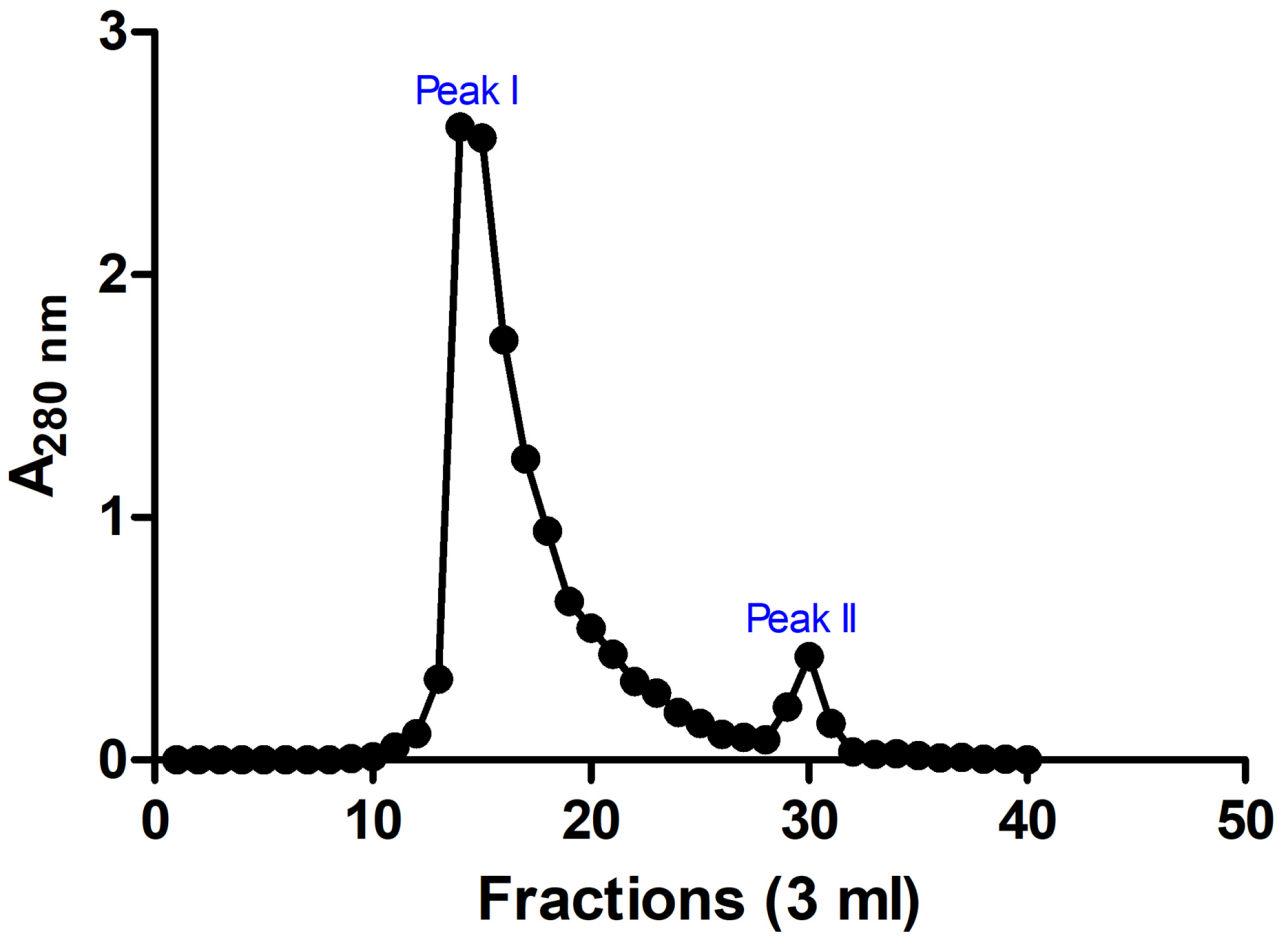
Chromatogram for fractions of gel filtration chromatography. Sephadex G-25 chromatographic resin was used and two prominent peaks (peaks I and II) were observed. Absorbance was measured at 280 nm. The image was rendered in *GraphPad 5.0* statistical package.

These SPE isolated peptides with molecular weight <5 kDa were further purified using Sephadex G-25 gel filtration chromatography to produce two peaks (Peak I and Peak II; Figure 3). The antimicrobial activity of the fractions in the peaks was evaluated. Results indicated that fractions of Peak I demonstrated high antimicrobial activity with a MIC of 0.31 ± 0.16 and 1.99 ± 0.13 μg/ml on *E. coli* and *S. aureus*, respectively, whereas no antimicrobial activity was detected in peak II (Table 1).

### Peptides Identification

**Figure 4:**
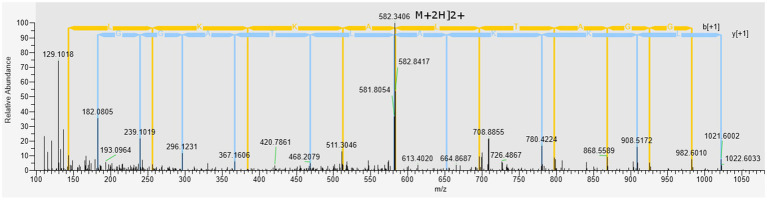
Mass spectrum (MS/MS) of the antimicrobial peptide (ACAP-I), from *C. gariepinus*. Representative MS^2^ data were obtained from a +2 parent ion with *m/z* 582.340 by HCD fragmentation and Orbitrap detection at 30.000 resolution. The *y*- and *b*- series of ions allows identification of the peptide AALKKALTAGGY from protein with accession number A0A7J6BGG9.

The Peak I fraction from gel filtration chromatography was subjected to LC–MS/MS at positive mode to uncover the molecular mass and amino acid sequences. A correlation of tandem mass spectral data of peptides with amino acid sequences in the compiled Catfish database using *Sequest HT*, positively identified 214 peptides in 126 proteins. Of the 214 peptides, some were predicted using *in silico* tools as antimicrobial while the majority were non-antimicrobial. First, a Discriminant Analysis machine learning revealed up to 40 of these peptides had antimicrobial probability score >0.5 (Supplementary File S1), in the range of 0.543–0.997 and hence were classified as AMPs, leaving 174 peptides as predicted as non-antimicrobial. The average antimicrobial score for the 40 AMPs was 0.797 with 12 peptides having higher antimicrobial scores (>0.9). Upon cross-validating, the 12 peptides using a more recently tuned rigorous Deep Learning approach, seven peptides stood out with an average antimicrobial score 0.749 (Table 2). Their antimicrobial score ranged from 0.561 to 0.905, with ACAP-I (amino acid residues: AALKKALTAGGY), from H15 domain-containing protein (Figure 4), demonstrating the highest antimicrobial score of 0.905.

**Table 2:** Physiochemical properties of most potent African Catfish Antimicrobial Peptides (ACAPs).

Seq. ID.	Sequences	L	Charge	MW	α-helix (%)	H (%)	AS	Processor protein
ACAP-I	AALKKALTAGGY	12	2	1163.68	58.30	75.00	0.905	H15 domain-containing protein
ACAP-II	AALKKALAAGGY	12	2	1133.67	66.67	83.33	0.898	H15 domain-containing protein
ACAP-III	GVASAPASGTGGFSFG	16	0	1369.64	0.00	75.00	0.768	Uncharacterized
ACAP-IV	KVSKVLHKAIL	11	3.5	1235.82	45.45	54.54	0.754	SERPIN domain-containing protein
ACAP-V	VVLGSGGVGKSAL	13	1	1143.67	0.00	76.92	0.684	Small monomeric GTPase
ACAP-VI	FGGAGVGKTVL	11	1	1005.57	0.00	81.81	0.677	ATP synthase subunit beta
ACAP-VII	IAIIPSKKLRNKIAG	15	4	1622.05	46.67	60.00	0.561	40S ribosomal protein

*L, peptide length; MW, molecular weight; H, hydrophobicity; and AS, antimicrobial score*.

### Sequence Characterization

**Figure 5:**
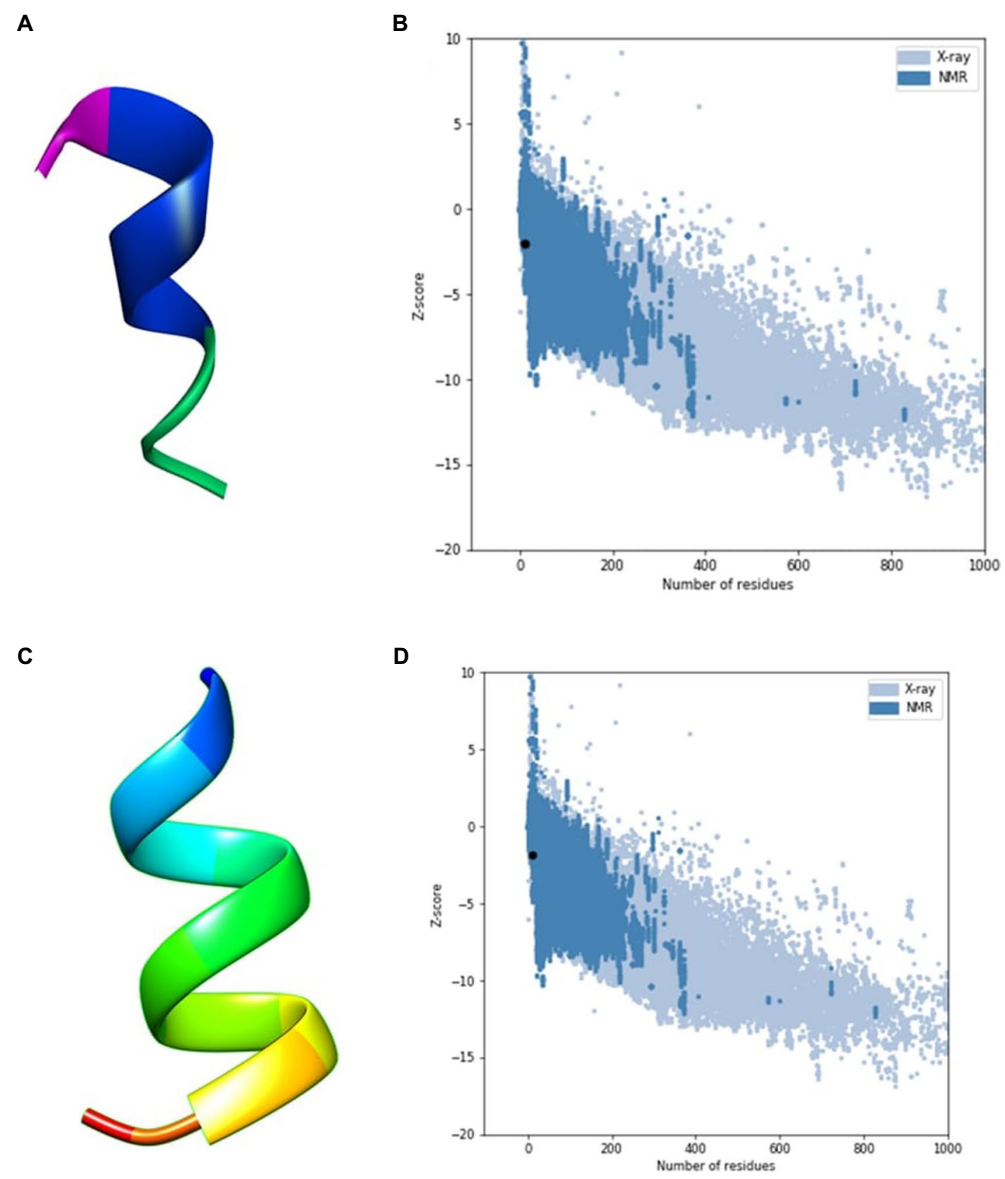
AWSEM and PEP-FOLD predicted peptide 3D structures of ACAP-I together with respective ProSA validation plots. **(A)** AWSEM-modeled ACAP-I predicted 3D structure, **(B)** AWSEM modeled ACAP-I ProSA *z*-score, **(C)** PEP-FOLD-modeled ACAP-I 3D structure, **(D)** PEP-FOLD-modeled ACAP-I ProSA *z*-score. AWSEM modeled ACAP-I and PEP-FOLD-modeled ACAP-I peptide had z-scores of −1.99 and −1.85, respectively, and were within the normal *z*-score of experimentally validated proteins.

An *in silico* analysis indicated that all the seven ACAPs were short. The length as determined by the LC–MS/MS ranged 11–16 amino acid residues. All the peptides demonstrated high hydrophobicity, with an average percentage hydrophobicity of 72.37%. The ACAP-II demonstrated the highest percentage hydrophobicity (83.33%) followed by peptide ACAP-VI (81.81%), with ACAP-IV having the lowest (54.54%). With exception to the neutral ACAP-III, the rest of identified AMPs were cationic with charge in the range of 1 to 4 (Table 2). Up to, four of these AMPs (ACAP-I, ACAP-II, ACAP-IV, and ACAP-VII) demonstrated α-helix structure conformation. This implies they can suitably be amphipathic. The molecular weight of the identified AMPs was low (1005.57–1622.05 Da). Three ACAP-I models were outputted by AWSEM and two by PEP-FOLD prediction tools. Just like in AWSEM, the PEP-FOLD model1 demonstrated lowest energy, and were therefore considered the best models. Upon subjecting the ACAP-I AWSEM and PEP-FOLD best 3D modeled structures to *ProSA* quality check, PEP-FOLD models demonstrated a better quality over AWSEM. Here, PEP-FOLD modeled ACAP-I_model1 demonstrated a higher similarity with those in the PDB database (*z* = −1.85) compared to AWSEM modeled (*z* = −1.99). Both the AWSEM and PEP-FOLD modeled structures displayed ribbon-like 3D structures with surface hydrophobic residues further confirming their possibility of forming α-helix structures (Figure 5).

## Discussion

In this study, we present a bioassay-guided fractionation and characterization of the AMPs, extracted from the acidic skin mucus of African catfish. The AMPs were sequentially isolated from the crude extract by ultrafiltration, SPE and then size exclusion chromatography. Thereafter, peptide identification was performed by mass spectrometry and the identified peptide sequences were further evaluated for potential antimicrobial properties. *In silico* tools were used for this purpose, including Deep-AmPEP30, EMBOSS PepStats, and Profile network prediction HeiDelberg (PHD) among others. The study utilized an ultrafiltration membrane to separate peptides of desired molecular weights and functional properties (Je et al., 2005). Besides, the membrane was capable of commanding the molecular weight distribution of the desired peptides (Kim et al., 1993). The additional strength of this study was hinged on maximal extraction of hydrophobic peptides, a key physiochemical parameter for any antimicrobial potential. This was due to cartridge’s solid-phase hydrophobic matrix optimal capturing of the hydrophobic peptides that are later recovered through organic solvent systems (Rana et al., 2018). The study focused on the three major lakes of Uganda (Lake Victoria, Lake Albert, and Lake Kyoga), and the antimicrobial activity varied across these lakes. This variation could be attributed to environmental disturbances like temperature, dissolved oxygen, pH, salinity and ecological niches that greatly influence the amount and quality of the mucus produced (Lebedeva, 1999; Subramanian et al., 2008; Nigam et al., 2012). Lately, considerable progress has been made in the search for bioactive compounds of aquatic origin (Giordano, 2020; Pooja et al., 2020). The need to develop new drug candidates to counter emerging and re-emerging diseases justifies the increased attention to this field (Quaglio et al., 2012). More so, the aquatic biodiversity abundance, coupled with the rapid advancement in processing technologies such as recombinant technology, solvent extraction, enzymatic treatment, solid-phase synthesis have boosted the search for novel biochemical compounds from such habitats. Furthermore, efficient downstream processing techniques like reverse phase High-Performance Liquid Chromatography (RP-HPLC) in union with extra analyzing equipment like ultra violet detector or mass spectrometer has spearheaded isolation and identification of novel bioactive compounds (Najafian and Babji, 2012; Jadaun et al., 2017).

As a result, aquatic-derived bioactive compounds have been reported to play a critical role to human health and nutrition. Notable examples include fish-derived clotting factors (Fomina et al., 2020), Seaweed antioxidants (Zerrifi et al., 2018), common water hyacinth, *Eichhornia crassipes* anticancer phytochemicals (Mtewa et al., 2021), anti-obesity polysaccharides from red algae (Yang et al., 2019), novel conopeptides from marine snails (Zhang et al., 2021), anti-inflammatory cembranoids from soft coral (Peng et al., 2020), novel AMPs from sea weed, *Porphyra yezoensis* (Jiao et al., 2019), anchovy fish-derived antioxidant peptides (Najafian and Babji, 2018), Marine-derived *Penicillium purpurogenum* anti-tumor metabolites (Teles et al., 2020), Salmon calcitonin anti-glycemic and anti-osteoporosis peptides (Chesnut et al., 2008), and fish-derived CF-14 AMPs (Li et al., 2019) among others. Despite, these tremendous contributions to drug leads, majority of the studies seem to focus much few highly valued species of marine habitats, with far limited emphasis on freshwater biochemical compounds.

The peak I AMPs in the present study demonstrated a high antimicrobial activity on both Gram-negative (*E. coli*) and Gram-positive (*S. aureus*) bacteria. This is invariable from previously reported fish AMPs like a 4.7 kDa LEAP-2 from Topmouth culter, *Erythroculter ilishaeformis* (Chen et al., 2020); 4.1 kDa CAP-IV from African catfish, *C. gariepinus* (Li et al., 2016); 2.2 kDa Pelteobagrin, from yellow catfish, *Pelteobagrus fulvidraco* (Su, 2011); 2.8 kDa SAMP-H1 from Atlantic salmon, *Salmo salar* (Luders et al., 2005) and 2.7 kDa Pleurocidin, from winter flounder, *Pleuronectes americanus* (Cole et al., 1997). This demonstrated that antimicrobial activity of AMPs may among other reasons, be attributed to their low molecular weight, as low molecular mass peptides (<5 kDa) exhibit high mobility and inhibitory activity (Enan et al., 1996). In this study, the molecular weight of ACAPs determined by LC–MS/MS ranges between 1.005 to 1.622 kDa. It is therefore not surprising that ACAPs demonstrated high antimicrobial scores (0.561–0.905).

Additionally, just like most AMPs: OVTp12 (Ma et al., 2020), Pelteobagrin (Su, 2011), Myxinidin (Subramanian et al., 2009), all the ACAPs except ACAP-III had a net positive charge of 1–4. This cationic property is due to the abundance of positively charged amino acid Arginine (Arg) and Lysine (Lys) residues in ACAPs. Several studies have demonstrated that net positive charge is one of the essential physiochemical parameters that determine the antimicrobial activity of any peptide (Bruston et al., 2007; Kim et al., 2018). This has been attributed to the fact that net positive charge on the peptides increases their affinity to initiate electrostatic interactions with the negatively charged phospholipid head groups on the outer surface of the microbial cell membrane, an asset to membrane penetration (Brogden, 2005). However, this charge should not exceed +9, as Therapeutic Index (TI), rapidly decreases beyond this value (Jiang et al., 2008). The charge of the identified peptides in this study were far below this threshold value rendering them as promising.

At the same time, ACAPs are rich in hydrophobic amino acids like Valine (Val), Glycine (Gly), Leucine (Leu), Isoleucine (Ile), Alanine (Ala), Phenylalanine (Phe), Tryptophan (Trp), and Proline (Pro). Hydrophobic peptides easily interact with cellular hydrophobic lipid bilayer, penetrate and cross the membrane (Bahar and Ren, 2013; Burton et al., 2013). In this study, SPE cartridges enhanced the recovery of such peptides. It is therefore not surprising that ACAPs all show more than 54% hydrophobicity, a value much higher than those in the previously reported peptides like Cruzioseptin (Cuesta et al., 2021), A15_B (Okella et al., 2020), OVTp12 (Ma et al., 2020) among others. Interestingly, four (ACAP-I, ACAP-II, ACAP-IV, and ACAP-VII) out of the seven identified ACAPs demonstrated α-helix structure conformation. This amphipathic physiochemical property has been reported as the most important for AMPs (Wang et al., 2019). This is because the polar region of the amphipathic peptide enhances association with the membrane through electrostatic interaction with the phospholipid charged head groups (Sato and Feix, 2006), as the non-polar region forms transient pores or channels through hydrophobic interactions with the non-polar region of the phospholipid bilayer, resulting into increased permeability and loss of barrier function of the bacterial cells (Sato and Feix, 2006; Mihajlovic and Lazaridis, 2012). It was beyond the scope of this study to reveal the cell membrane distraction level of ACAPs.

## Conclusion

This study identified seven potential AMPs from the African catfish’s skin mucus. These AMPs are new potential antibiotic candidates of application to food and pharmaceutical industries. We recommend further studies to explore the drug-likeness and possible mode of action of promising individual African Catfish Antimicrobial peptide (ACAP).

## Data Availability Statement

The datasets presented in this study can be found in online repositories. The names of the repository/repositories and accession number(s) can be found at: ProteomeXchange – PXD029193 https://www.ebi.ac.uk/pride/archive?keyword=PXD029193.

## Ethics Statement

The animal study was reviewed and approved by Mbarara University of Science (Approval No. 22/11-18) and Technology Research Ethics Committee and Uganda National Council for Science and Technology (Approval Reference No. HS449ES).

## Author Contributions

HO and SOc designed and implemented the study. DV, CN, JA, CA, HI, JN, CK, PO, and HO performed the experiments. HO, BK, DV, AM, SOd and TK performed data analysis. All authors participated in writing and proof reading the manuscript and approved the final manuscript for publication.

## Funding

This research was supported by the International Foundation for Science (A_6226-1), Stockholm, Sweden, through a grant to HO.

## Conflict of Interest

The authors declare that the research was conducted in the absence of any commercial or financial relationships that could be construed as a potential conflict of interest.

## Publisher’s Note

All claims expressed in this article are solely those of the authors and do not necessarily represent those of their affiliated organizations, or those of the publisher, the editors and the reviewers. Any product that may be evaluated in this article, or claim that may be made by its manufacturer, is not guaranteed or endorsed by the publisher.

## Footnotes

^1^ https://aps.unmc.edu/

^2^ http://www.camp3.bicnirrh.res.in/

^3^ https://prosa.services.came.sbg.ac.at/prosa.php

^4^ https://www.rcsb.org/

^5^ https://data.humdata.org/dataset/uganda-administrative-boundaries-admin-1-admin-3

## Supplementary Material

The Supplementary Material for this article can be found online at: https://www.frontiersin.org/articless/10.3389/fmicb. 2021.794631/full#supplementary-material
