# Supplementary material for: Identification of Antimicrobial Peptides Isolated From the Skin Mucus of African Catfish, Clarias gariepinus (Burchell, 1822)
Source: Front Microbiol. 2021 Dec 20;12:794631. doi: 10.3389/fmicb.2021.794631 (PMC8721588; doi:10.3389/fmicb.2021.794631)
Supplement: Supplementary file 1 [file Table_1.DOC]

***Supplementary File 1***

| **S/No** | **Seq. Processor ID.** | **Sequences** | **Antimicrobial score** |
| --- | --- | --- | --- |
| 1 | A0A7J5ZUT8_2 | GAGGGFGAGGGFGSGGGFGGGFG | 0.997 |
| 2 | A0A7J5ZUT8_1 | GAGGGFGAGGGFGSGGGFG | 0.992 |
| 3 | A0A5N5LC08 | KVSKVLHKAIL | 0.975 |
| 4 | A0A7J6ATJ1_1 | AALKKALAAGGY | 0.963 |
| 5 | A0A7J6ALK6_3 | FGGAGVGKTVL | 0.954 |
| 6 | A0A2D0RRS1 | AGGGAGGGGGFGFGGGF | 0.951 |
| 7 | A0A7J6AHG0 | IAIIPSKKLRNKIAG | 0.936 |
| 8 | A0A7J6AA08_1 | VEDFKKKYEDEINKRAAAENE | 0.929 |
| 9 | A0A556UXS0 | VVLGSGGVGKSAL | 0.920 |
| 10 | A0A5N5KSD9 | GVASAPASGTGGFSFG | 0.917 |
| 11 | A0A7J6BGG9 | AALKKALTAGGY | 0.911 |
| 12 | A0A5N5PKX8 | INLRIEHEVAGGIAGLRAAMEALVV | 0.908 |
| 13 | A0A2D0RAQ3 | GAGGAGLALGGGGGAGAL | 0.889 |
| 14 | A0A5N5JEQ1_1 | VVINPYKNLPIY | 0.884 |
| 15 | A0A556TWZ9 | LGSGGLGLGLGSGGL | 0.875 |
| 16 | A0A5N5NK88 | IKQIPRILGPGLN | 0.832 |
| 17 | S4V1J6_1 | IINMKPPAISQ | 0.826 |
| 18 | A0A7J6AA08_3 | VEDFKKKYEDEINKRAAAENEF | 0.824 |
| 19 | A0A2D0S7F3_2 | ILDNGANGKIFV | 0.814 |
| 20 | A0A7J5ZJV7 | LAAGGIYHPRL | 0.796 |
| 21 | A0A5N5PZE3 | AGGGAGGGGGGGGGGGGGGGGGGGL | 0.778 |
| 22 | A0A556U3B9_1 | FTNHNGTGGKSIYGNKF | 0.772 |
| 23 | A0A556V9V8 | VVINQKGIDPFSL | 0.761 |
| 24 | A0A5N5PXY6 | GGVGGGGGGGGGGGGGGGSGGGTI | 0.759 |
| 25 | A0A556V121 | AAIEPSRGRPAGLGDALVPAAAGA | 0.754 |
| 26 | A0A2D0RPR7 | LAGRSGGAGTAGGGGAI | 0.752 |
| 27 | A0A5N5NJW0_2 | IFHSFGGGTGSGF | 0.750 |
| 28 | A0A7J5ZSW9_2 | IFHSFGGGTGSGF | 0.750 |
| 29 | W5UM08 | AILGGAKVKDKIQL | 0.702 |
| 30 | A0A556UYJ6_4 | IKVDKGVVPLAGTNG | 0.691 |
| 31 | Q90YT2_1 | LKVSKDKRALKF | 0.684 |
| 32 | A0A2D0T815 | VGGGGGGGGSGGGGFPGNGGGS | 0.682 |
| 33 | W5UKQ5 | LPGSSSSGFAGSGF | 0.671 |
| 34 | A0A2D0S7F3_1 | ILDNGANGKIF | 0.668 |
| 35 | A0A2D0SXG7 | KSVGGGGGGGVGGGGGGGGA | 0.656 |
| 36 | A0A2D0QLM1_1 | VYKVLKQVHPDTGISSKA | 0.621 |
| 37 | A0A5N5LUK7_1 | VFHSFGGGTGSGF | 0.618 |
| 38 | A0A5N5KZN5 | GKTAGWNIPIGTL | 0.591 |
| 39 | A0A5N5PRH7 | FKTQFGGGKTTGF | 0.577 |
| 40 | A0A5N5LBM3 | IGGISGGGATSRL | 0.543 |
